# Testosterone Deficiency, Weakness, and Multimorbidity in Men

**DOI:** 10.1038/s41598-018-24347-6

**Published:** 2018-04-12

**Authors:** Mark D. Peterson, Aleksandr Belakovskiy, Ryan McGrath, Joshua F. Yarrow

**Affiliations:** 1 Department of Physical Medicine & Rehabilitation, Michigan Medicine, University of Michigan, Ann Arbor, USA; 2 Department of Family Medicine, Michigan Medicine, University of Michigan Research Service, Ann Arbor, USA; 3 Malcom Randall Department of Veterans Affairs Medical Center, North Florida/South Georgia Veterans Health System, Gainesville, USA; 4 Division of Endocrinology, Diabetes, and Metabolism, University of Florida College of Medicine, Gainesville, USA

## Abstract

The purposes of this study were to evaluate the association between total testosterone (TT) deficiency and weakness on multimorbidity in men. Analyses were performed to examine the prevalence of multimobidity among young, middle-aged, and older men, with and without testosterone deficiency. Multivariate logistic models were also used to determine the association between age-specific TT tertiles and multimorbidity, adjusting for key sociodemographic variables, as well as a secondary analysis adjusted for grip strength. Multimorbidity was more prevalent among men with testosterone deficiency, compared to normal TT in the entire group (36.6% vs 55.2%; p < 0.001); however, differences were only seen within young (testosterone deficiency: 36.4%; normal TT: 13.5%; p < 0.001) and older men (testosterone deficiency: 75.0%; normal TT: 61.5%; p < 0.001). Robust associations were found between the age-specific low-TT (OR: 2.87; 95%CI: 2.14–3.83) and moderate-TT (OR: 1.67; 95%CI: 1.27–2.20) tertiles (reference high-TT) and multimorbidity. Secondary analysis demonstrated that both low TT (OR: 1.82; 95%CI: 1.29–2.55) and moderate-TT (OR: 1.31; 95%CI: 1.01–1.69) were associated with multimorbidity, even after adjusting for obesity (OR: 1.75; 95%CI: 1.07–2.87) and NGS (OR: 1.21 per 0.05 unit lower NGS). Low TT and weakness in men were independently associated with multimorbidity at all ages; however, multimorbidity was more prevalent among young and older men with testosterone deficiency.

## Introduction

The prevalence of total testosterone (TT) deficiency in men increases with age^1,2^, and is associated with several deleterious effects to the musculoskeletal system including osteopenia and sarcopenia^3^, as well as with higher rates of all-cause mortality^4–8^. Recent studies have also suggested that testosterone deficiency is independently and robustly associated with various obesity-related chronic diseases in men, including type 2 diabetes and cardiovascular disease^9–11^. However, there is uncertainty as to what constitutes optimal physiological levels of TT among men across different age categories, and to the effects that varying TT levels have on disease risk^12^. This debate is partially fueled by inconsistent findings in the literature and differences across expert clinical recommendations, as well as an incomplete understanding of the underlying mechanisms and temporal sequence of events leading to and stemming from testosterone deficiency. For example, the authors of a well-known systematic review assert that although testosterone deficiency is robustly associated with obesity, insulin resistance, low high-density lipoprotein (HDL) cholesterol and elevated triglycerides, low-density lipoprotein (LDL) cholesterol, and plasminogen activator type 1, there is insufficient evidence to demonstrate a causal role of endogenous testosterone deficiency on coronary artery disease^13^-the leading cause of preventable death among men in the U.S^14^.

Harmonized reference ranges for TT were recently established by combining cohort studies in the U.S. and Europe^15^. That investigation effectively defined “normal ranges” for TT levels in young healthy men, which may help limit misdiagnosis of testosterone deficiency. However, those ranges were derived from non-nationally representative pooled data across cohorts that do not reflect the growing, ethnically-diverse U.S. population, and in which individuals with several morbid conditions were excluded. Therefore, the primary purpose of this study was to evaluate the association between TT deficiency and prevalent chronic multimorbidity in a large, population-representative sample of U.S. men. We also sought to determine a dose-response trend by examining the effect of age-specific TT tertiles on multimorbidity.

## Research Design and Methods

### Study Population

The National Health and Nutrition Examination Survey (NHANES) is a program of studies designed to assess the health and nutritional status of adults and children in the United States. The 2011–2012 NHANES survey was chosen based on the wealth of relevant information pertaining to chronic diseases, total testosterone, and direct measures of muscle strength capacity. The analyses were performed in 2016–2017. This survey is unique in that it combines interviews and physical examinations; however, not all participants in the physical exam are included. Of the 2,399 men in the 2011–2012 NHANES survey who were at least 20 years old, 2,161 had complete data for (1) demographic and anthropometric information; (2) valid questionnaire data pertaining to chronic disease diagnoses (e.g., diabetes); (3) the necessary blood samples obtained for serum TT determination; (4) valid strength data from a handgrip dynamometer; and (5) the necessary examination or laboratory assessments for cardiometabolic disease risk factors. Ethical approval was obtained through the National Center for Health Statistics (NCHS) Research Ethics Review Board (Protocol #2011-17). All procedures followed were in accordance with the ethical standards of the NCHS Research Ethics Review Board, and with the Helsinki Declaration, and informed consent was obtained from all patients included in the study.

### Demographic and Anthropometric Factors

Socio-demographic characteristics were self-reported during the in-home interview. Age was used as a categorical variable as (1) 20–39.9 years, (2) 40–59.9 years, and (3) ≥60 years old. Race/ethnicity was categorized as: (1) non-Hispanic white, (2) non-Hispanic black, (3) Mexican American or other Hispanic, and (4) Other-including multi-racial. Education was categorized as: (1) less than high school graduate, (2) high school graduate/general educational development (GED) or equivalent, and/or some college or Associate’s degree, and (3) college graduate or above. Marital status was dichotomized as married or not married. Annual household income was categorized as: (1) ≤$24,999, (2) $25,000-$54,999, (3) $55,000-$74,999, and (4) ≥$75,000.

Weight was measured using a digital Toledo scale (Mettler-Toledo International, Inc., Columbus, OH), and participants only wore an underwear gown and foam slippers. Height was measured using a fixed stadiometer. BMI was calculated as weight in kilograms divided by height in meters squared (kg/m^2^). Standard categories were applied to determine if each participant was obese (≥30 kg/m^2^). Individuals with BMI <18.5 kg/m^2^ were excluded, due to the known association between underweight status and chronic disease risk^16^. Waist circumference was measured to the nearest 0.1 cm at the level of the iliac crest. Standard cut points for abdominal obesity in men (>102 cm) were used.

### Self-Reported Health Conditions

The NHANES collects self-reported primary health conditions by asking whether the participant has “ever been told by a doctor or health professional” that he or she has a health condition. For this study, the prevalence of 7 self-reported chronic conditions was evaluated, including diagnosis of: type 2 diabetes, arthritis, cardiovascular disease (congestive heart failure, coronary heart disease, and/or angina), stroke, pulmonary disease (emphysema), hypertension, and clinical depression. Clinical depression was dichotomized as a score of ≥10 on the 9-item Patient Health Questionnaire. Scores of 10 or higher have high sensitivity and specificity for identifying major depression in a primary care setting^17^.

### Examination and Laboratory Cardiometabolic Abnormalities

In addition to the aforementioned self-reported health conditions, participants were tested on routine cardiometabolic parameters with physical examination and laboratory assessments. Detailed descriptions of the laboratory protocols are provided in the NHANES Laboratory Procedures Manual (https://wwwn.cdc.gov/nchs/data/nhanes/2011-2012/manuals/2011-12_Laboratory_Procedures_Manual.pdf). Resting systolic and diastolic blood pressures were measured three to four times with a mercury sphygmomanometer by trained staff. Fasting and non-fasting measures of HDL-cholesterol, triglycerides, and glucose were measured. Non-fasting serum measures of glycated hemoglobin (HbA1c) were included as a diagnostic test for untreated diabetes, which reflects average plasma glucose for the previous ~three-months.

The diagnostic criterion for diabetes was defined as self-reported diabetes, elevated fasting glucose (≥126 mg/dL), *or* HbA1c values ≥6.5% (≥48 mmol/mol)^18^. Participants with diabetes that were being treated with only insulin alone were excluded, as they were considered likely to have type 1 diabetes. Hypertension was defined as self-reported history of hypertension (i.e., physician diagnosis on 2 or more different visits), a systolic blood pressure ≥140 mmHg, *or* a diastolic blood pressure ≥90 mmHg. Hypertriglyceridemia was determined as ≥150 mg/dL, and low HDL-cholesterol was determined as <40 mg/dL and <50 mg/dL for men and women, respectively.

### Multimorbidity

Multimorbidity was defined as the presence of at least two chronic conditions among a list of the nine aforementioned self-reported chronic diseases or examination/laboratory cardiometabolic abnormalities (i.e., diabetes, hypertension, arthritis, cardiovascular disease, stroke, emphysema, hypertriglyceridemia, low-HDL cholesterol, and clinical depression). These conditions were selected in accordance with guidance from the literature pertaining to older adults and adults with disabilities^19–21^, and availability and reliability of the diagnosis within our clinical data.

### Exposure Variable

TT levels were measured in serum using isotope dilution liquid chromatography tandem mass spectrometry by the National Center for Environmental Health, Centers for Disease Control and Prevention. This method employed LLE extractions of serum to isolate the steroid. Stable Isotope-labeled testosterone is used as an internal standard to correct for sample recovery during the sample preparation process. The assay imprecision is 5% and the method is traceable to an established higher-order testosterone reference method (see: https://www.cdc.gov/nchs/data/nhanes/nhanes_11_12/tst_g_met.pdf). Testosterone deficiency was categorized as <300 ng/dL (10.4 nmol/L)^22^. Age-category-specific TT tertiles were created for use in the logistic regression analyses, as the: (1) highest TT tertile (ages 20–39.9 years: >488 ng/dL; ages 40–59.9 years: >440 ng/dL; and ≥60 years: >433 ng/dL); (2) medium TT tertile (ages 20–39.9 years: 347–488 ng/dL; ages 40–59.9 years: 291–440 ng/dL; and ≥60 years: 290–433 ng/dL); and lowest TT tertiles (ages 20–39.9 years: <347 ng/dL; ages 40–59.9 years: <291 ng/dL; and ≥60 years: <290 ng/dL).

### Covariate

Muscle strength was assessed using a hydraulic handgrip dynamometer (Takei Digital Grip Strength Dynamometer, Model T.K.K.5401). Detailed descriptions of the protocol are provided in the NHANES Muscle Strength/Grip Test Procedure Manual^23^. A trained examiner explained and demonstrated the protocol to each participant, then adjusted the grip size of the dynamometer to the participant’s hand size, and asked the participant to squeeze the dynamometer for a practice trial. Participants were randomly assigned to start the test with the dominant or non-dominant hand, and asked to squeeze the dynamometer with maximal effort, exhaling while squeezing. Each hand was tested three times. Grip strength was normalized (NGS) as strength per body mass.

### Statistical analysis

All statistical analyses were performed using SAS 9.3 (SAS Institute, Cary, NC). NHANES employs a multistage sampling design. Sample weights were used to adjust for oversampling, survey nonresponse, and post-stratification. Further, we took into account subsample weights since we conducted analyses on persons with non-fasting glucose measure. These weights were used to produce unbiased estimates. To obtain correct variance estimation, information on strata and primary sampling unit (PSU) were also utilized. Differences in these characteristics across age categories and for testosterone deficiency were tested using linear regression (proc surveyreg) and logistic regression (proc surveylogistic) for continuous and categorical variables respectively, after creating appropriate categories and dummy coding for each. Partial correlation statistics were conducted to examine the association between TT and NGS, while adjusting for age, race/ethnicity, education and household income.

To assess the odds of multimorbidity in the entire sample, we utilized the univariate and multivariate logistic regression modeling approaches. For model 1, only age-category-specific TT tertiles were entered as the primary exposure variable (reference: High TT tertile). For model 2, further adjustments for known demographic covariates, including age categories, race, education, annual income, and marital status, were included in each model. Lastly, in order to examine whether the association between TT and multimorbidity was explained in whole or part by obesity or NGS capacity, a third model was performed including (1) age-category-specific TT tertiles; (2) all sociodemographic variables; (3) obesity status; and (4) NGS (with units set to 0.05).

### Data availability

All data are publically available and were obtained through the CDC’s National Health and Nutrition Examination Survey website: https://wwwn.cdc.gov/nchs/nhanes/continuousnhanes/default.aspx?BeginYear = 2011.

## Results

The descriptive data of all 2,399 men are presented across age categories in Table 1. Obesity, waist circumference, and most cardiometabolic health parameters were significantly lower among young men (p < 0.001); however, in many cases, middle-aged men had worse cardiometabolic profiles than young *and* older men. Absolute grip strength and NGS were both significantly lower across higher age categories (p < 0.001). Moreover, TT was highest among young men (p < 0.001); however, there were no differences in TT between middle-aged and older men.

**Table 1:** Descriptive and health characteristics of the young (20–39.9 years), middle-aged (40–59.9 years) and older (≥60 years) men; Mean (standard deviation) or percentages.

	Age 20–39.9 years	Age 40–59.9 years	Age ≥60 years
	*n* = *864*	*n* = *766*	*n* = *769*
Age, years	29.12 (5.85)	49.33 (5.73)*	69.80 (6.99)^‡§^
Body Mass Index (BMI), kg/m^2^	27.95 (6.22)	28.93 (6.09)*	28.34 (5.25)
Obesity (BMI >30), %	29.8	35.2*^‡^	32.3^§^
Waist Circumference (WC), cm	95.25 (16.57)	101.59 (15.55)*	103.59 (14.16)^‡§^
Abdominal Obesity (WC >102 cm), %	29.3	43.7*	50.2^‡§^
Grip Strength, kg	49.81 (9.34)	46.31 (8.48)*	38.39 (8.79)^‡§^
^a^Normalized Grip Strength (NGS)	0.60 (0.13)	0.54 (0.12)*	0.47 (0.11)^‡§^
Total Testosterone (TT), ng/dL	434.88 (173.78)	381.40 (160.69)*	383.33 (183.90)^§^
Glycated Hemoglobin (HbA1c), %	5.45 (0.88)	5.93 (1.25)*	6.16 (1.23)^‡§^
Glucose, mg/dL	100.44 (24.84)	114.93 (43.47)*	120.44 (38.07)^‡§^
Insulin, μU/mL	12.92 (11.19)	14.74 (15.27)*	13.55 (11.78)
Triglycerides, mg/dL	123.33 (79.70)	148.18 (84.24)*^‡^	123.34 (67.68)
Total Cholesterol, mg/dL	182.43 (37.05)	197.08 (41.24)*^‡^	179.65 (42.17)
HDL-Cholesterol, mg/dL	47.78 (12.20)^§^	46.83 (12.84)^‡^	49.32 (14.43)
LDL-Cholesterol, md/dL	112.81 (33.79)^§^	119.70 (35.69)*^‡^	105.50 (35.91)
Systolic Blood Pressure, mmHg	119.08 (11.76)	125.46 (17.00)*	133.75 (19.62)^‡§^
Diastolic Blood Pressure, mmHg	71.90 (10.38)	77.39 (10.53)*^‡^	71.79 (11.09)

Abbreviations: BMI-body mass index; WC-waist circumference; NGS-Normalized Grip Strength; TT-Total Testosterone; HbA1c-Glycated Hemoglobin; HOMA-Homeostasis Model of Assessment; HDL-high density lipoprotein; LDL-low density lipoprotein.

^a^Grip strength in kg divided by body mass in kg.

*Significant difference between ages 20–39.9 years and 40–59.9 years (p < 0.01): Denoted as group with higher risk.

^‡^Significant difference between ages 40–59.9 years and ≥60 years (p < 0.01): Denoted as group with higher risk.

^§^Significant difference between ages 20–49.9 years and ≥60 years (p < 0.01): Denoted as group with higher risk.

### Chronic Disease Risk Factors and Multimorbidity

For the full sample, the mean number of chronic disease risk factors was 1.5 ± 1.3, with hypertension as the most prevalent individual condition (48.6%), followed by hypertriglyceridemia (28%), low HDL-C (26.8%), arthritis (19.7%), diabetes (17.9%), clinical depression (7.4%), CVD (7.2%), stroke (4%), and pulmonary disease (2.5%).

The prevalence of multimorbidity was 41.3% for all men; however, there was a significant age association (p < 0.001). For young, middle-aged, and older men, the prevalence of multimorbidity was 17.4%, 44.2%, and 65.3%, respectively. Of the 502 total possible combinations of multimorbidity, we found 58 unique multimorbidity combinations, and the most prevalent combinations were: (a) hypertension and arthritis (n = 107); (b) hypertension and low HDL-C (n = 103); (c) diabetes and hypertension (n = 86); (d) hypertension and hypertriglyceridemia (n = 50); hypertriglyceridemia and low HDL-C (n = 47); and diabetes, hypertension, and arthritis (n = 42).

### Testosterone Deficiency

**Figure 1:**
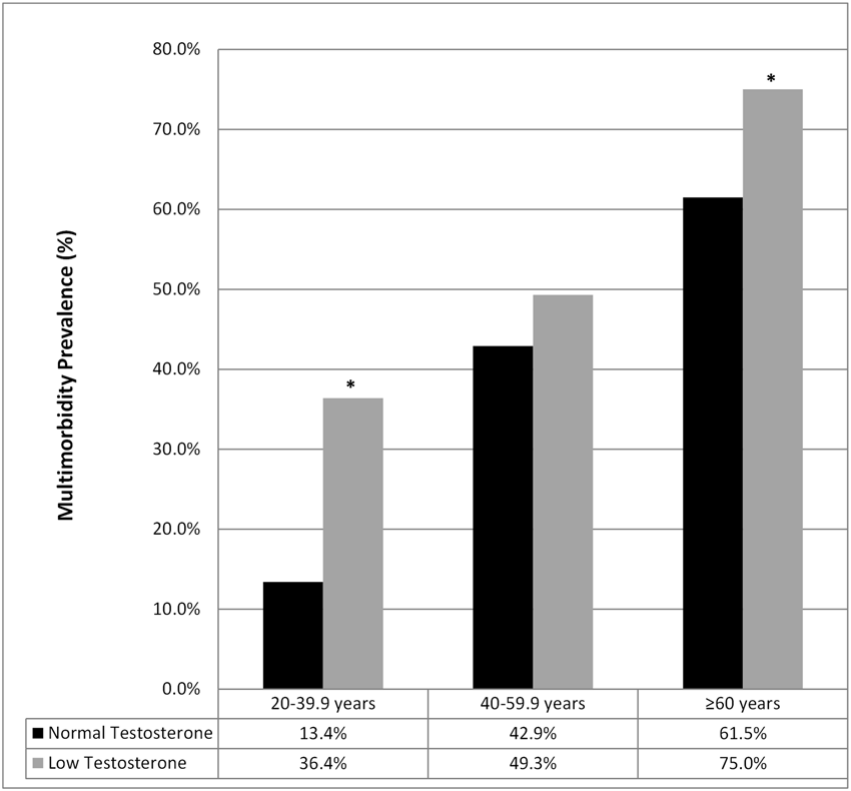
Mulitmorbidity prevalence between men with low TT versus normal TT, across age categories.

Prevalence of testosterone deficiency (<300 ng/dL [10.4 nmol/L]) was 30.8% for the entire sample, and 22.6%, 35.8%, and 34.6% for young, middle-aged, and older men, respectively. Significant higher prevalences of individual chronic diseases were found between men with testosterone deficiency as compared to men with normal TT (Table 2). Further, multimorbidity was significantly more prevalent among men with testosterone deficiency, compared to normal TT in the entire group (36.6% vs 55.2%; p < 0.001); however, differences were only seen within the younger (testosterone deficiency: 36.4%; normal TT: 13.5%; p < 0.001) and older men (testosterone deficiency: 75.0%; normal TT: 61.5%; p < 0.001) (Fig. 1).

**Table 2:** Prevalence (%) of chronic conditions/diseases between men with testosterone deficiency (<300 ng/dL [10.4 nmol/L])) versus those with normal TT.

Prevalence (%)	Age 20–39.9 years		Age 40–59.9		Age ≥ 60 years	
	Normal TT (≥300 ng/dL)	TT Deficiency (<300 ng/dL)	Normal TT (≥300 ng/dL)	TT Deficiency (<300 ng/dL)	Normal TT (≥300 ng/dL)	TT Deficiency (<300 ng/dL)
	*n* = *669*	*n* = *195*	*n* = *490*	*n* = *276*	*n* = *501*	*n* = *268*
Obesity (BMI >30)	22.8	55.7*	30.0	48.0*	25.8	45.9*
Abdominal Obesity (WC >102 cm)	21.5	57.1*	37.1	59.5*	42.9	64.9*
Diabetes	3.1	7.7*	15.9	26.5*	27.9	41.8*
Arthritis	3.6	4.1	18.2	18.1	36.3	44.4*
Cardiovascular Disease	0.3	2.1*	5.5	2.9	15.2	20.2*
Stroke	0.2	0.1	2.9	1.5	10.2	7.8
Pulmonary Disease	0.0	1.0	2.0	1.8	3.8	7.5*
Hypertension	18.4	38.5*	49.6	50.7	73.0	80.0*
Clinical Depression	5.5	11.5*	8.7	8.0	6.2	6.7
Low HDL-Cholesterol	20.2	42.1*	25.1	39.1*	20.2	35.1*
Hypertriglyceridemia	17.9	48.0*	33.6	50.1*	20.4	32.2*
*Multimorbidity*	*13.5*	36.4*	*42.9*	*49.3*	*61.5*	75.0*

^*^Significant difference between subjects with and without Testosterone deficiency, within the same category.

**Figure 2:**
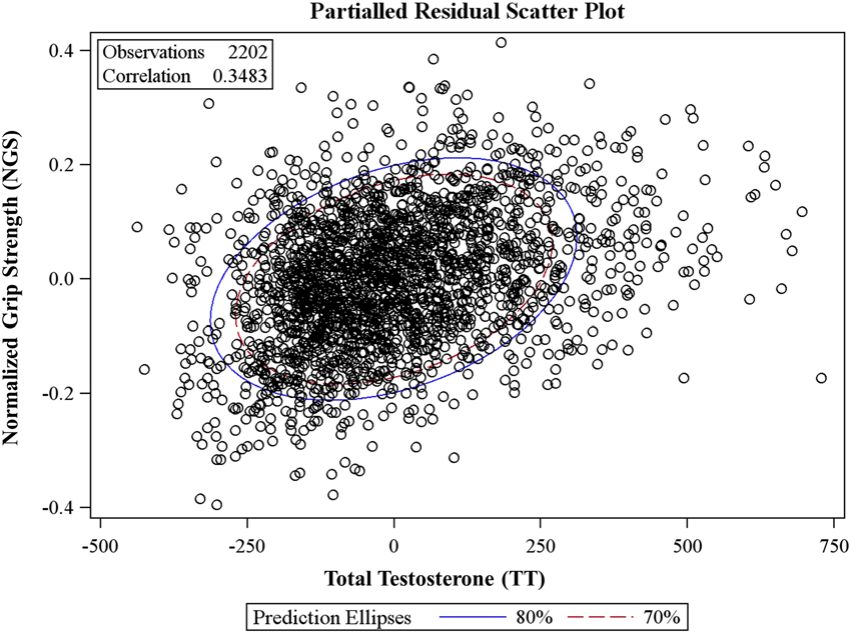
Partial residual scatter plot revealing the correlations between total testosterone and normalized strength capacity after adjustment for age, race/ethnicity, education, and income.

Both TT and NGS were robustly associated with multimorbidity (p < 0.001). Secondary analysis demonstrated that TT was significantly correlated to NGS (r = 0.35; p < 0.001) (Fig. 2), even after adjusting for age, race/ethnicity, income and education. Unadjusted and adjusted logistic models revealed a robust association between the age-specific low-TT and moderate-TT tertiles (reference high TT tertiles) and multimorbidity (Table 3). In the final multiple logistic model, both low TT and moderate-TT tertiles were still significantly associated with multimorbidity even after adjusting for NGS (Table 4)

**Table 3:** Univariate and sociodemographic-adjusted logistic regression models for multimorbidity in adult men.

Model Predictor(s)	Odds Ratio	95% CL	Pr > ChiSq
*Unadjusted Model*			
^a^Total Testosterone Tertiles (Ref: High Testosterone)			
Low Testosterone	2.18	1.71–2.79	<0.001
Medium Testosterone	1.39	1.09–1.77	0.01
*Model 2: Sociodemographic Adjusted*			
Age Category (Ref: 20–399 years)			
Age 40–59.9 years	4.52	3.19–6.42	<0.001
Age ≥ 60 years	9.19	6.87–12.31	<0.001
Race/ethnicity (Ref: Non-Hispanic White)			
Non-Hispanic black	0.98	0.76–1.26	0.86
Hispanic or Mexican American	1.04	0.79–1.37	0.79
Other, including multi-racial	0.96	0.61–1.49	0.85
Education Level (Reference: College Graduate)			
<High School Graduate	1.67	0.89–3.14	0.11
Some College	1.43	0.79–2.61	0.24
Marital Status (Reference: Unmarried)	1.09	0.76–1.56	0.66
Income Level (Reference: ≥$75,000)			
<$25,000	2.11	1.37–3.25	0.01
$25,000–$54,999	1.22	0.79–1.90	0.37
$55,999–$74,999	1.32	0.89–1.95	0.16
^a^Total Testosterone Tertiles (Ref: High Testosterone)			
Low Testosterone	2.87	2.14–3.83	<0.001
Medium Testosterone	1.67	1.27–2.20	<0.001

^a^Denotes age-category specific Total Testosterone Tertiles.

**Table 4:** Multiple logistic regression models for independent predictors of multimorbidity including the effect of obesity and NGS.

Model Predictor(s)	Odds Ratio	95% CL	Pr > ChiSq
Age Category (Ref: 20–399 years)			
Age 40–59.9 years	3.62	2.43–5.40	<0.001
Age ≥60 years	6.23	4.07–9.54	<0.001
Obesity (BMI ≥30)	1.75	1.07–2.87	0.03
Race/ethnicity (Ref: Non-Hispanic White)			
Non-Hispanic black	1.08	0.79–1.46	0.63
Hispanic or Mexican American	0.97	0.71–1.33	0.85
Other, including multi-racial	1.13	0.75–1.68	0.57
Education Level (Reference: College Graduate)			
<High School Graduate	1.52	0.76–3.02	0.23
Some College	1.27	0.70–2.30	0.43
Marital Status (Reference: Unmarried)	1.29	0.82–2.03	0.27
Income Level (Reference: ≥$75,000)			
<$25,000	2.41	1.53–3.80	<0.001
$25,000–$54,999	1.24	0.79–1.94	0.36
$55,999–$74,999	1.21	0.77–1.89	0.41
^a^Total Testosterone Tertiles (Ref: High Testosterone)			
Low Testosterone	1.82	1.29–2.55	<0.001
Medium Testosterone	1.31	1.01–1.69	0.04
Normalized Grip Strength	1.21	1.08–1.35	<0.001

^a^Denotes age-category specific Total Testosterone Tertiles.

*OR and 95%CL per each 0.05 unit lower.

## Discussion

The principal findings of this study were that TT deficiency was robustly and independently associated with multimorbidity in a large population-representative sample of U.S. men. Moreover, there was strong evidence supporting a dose-response trend for TT and multimorbidity risk. Specifically, the lowest age-category-specific TT tertile and the middle age-category-specific TT tertile were associated a >3-fold and nearly 75% higher multimorbidity risk, when compared to the highest age-category-specific tertiles of TT. Our results support the findings of several other large population-based prospective studies that indicate increased all-cause mortality and cardiovascular risks in both young and older men within the lowest ranges of TT, in comparison to men in the highest^5–7,24^. The dose-response trend we observed remained significant even after adjusting for grip strength, a known, robust predictor of chronic disease and early mortality in men^25,26^. Similarly, others have reported reduced all-cause and cardiovascular mortality accompany each 1 standard deviation, or 1 nmol/L, incremental increase in TT, even for men in the eugonadal range^11,24,27^. Further, a large meta-analysis of observational studies (n = 16,184 men) reported a 55% higher all-cause mortality (RR: 1.55; 95% CI: 1.28 to 1.88) for studies with baseline TT ≤ 487 nd/dl^7^, suggesting a threshold for increased mortality that is very near our TT cutoffs for the highest age-category-specific tertiles.

We have also uncovered interesting age disparities in testosterone deficiency and multimorbidity prevalence in our population-representative sample of U.S. men. Specifically, there was a much lower median TT in the youngest age category of men from our study (419 ng/dL), as compared to previous clinical cohort studies which harmonized TT reference ranges from healthy, non-obese men of the same age (533 and 529 ng/dL)^15^. This is very likely due to (1) the previous cohort populations, which were mainly men identified as white; (2) exclusion of ~5% of men who exhibited pituitary, testicular or adrenal disease, or who used medications that affect sex-steroid production from the European Male Aging Study, and those with BMI >30 kg/m^2^ from the Belgium Siblings Study of Osteoporosis^15^; and (3) the lack of adjustment for important sociodemographic factors such as race/ethnicity, income, education, and marital status. Moreover, the recently published harmonized 2.5^th^, 5^th^, 50^th^, 95^th^, and 97.5^th^ percentiles were 264, 303, 531, 852, and 916 ng/dL, respectively, for healthy, non-obese men aged 19–39 years. Currently used cutoffs to diagnose TT deficiency (<300 ng/dL [10.4 nmol/L]) would therefore correspond with the 5^th^ percentile of the reference range; however, in our study the 5^th^ percentile among young men aged 20–39 corresponded with a TT of 182 ng/dL.

Moreover, multimorbidity was significantly more prevalent among men with testosterone deficiency, compared to normal TT in the entire group (36.6% vs 55.2%); and yet, these differences seem to have been largely driven by differences in multimorbidity for young and older men. Despite strong evidence of worse chronic disease risk profiles among middle aged men with testosterone deficiency, as compared to middle-aged men with normal TT, the differences in multimorbidity were not statistically different. Collectively, this demonstrates the importance of screening TT among young men, particularly those with existing obesity, diabetes, cardiovascular disease, hypertension, clinical depression, low HDL cholesterol, or hypertriglyceridemia, as each of these conditions were significantly higher in those with testosterone deficiency. This is in accordance with the 2016 American Association of Clinical Endocrinologists and American College of Endocrinology (AACE/ACE) Comprehensive Clinical Practice Guidelines for Medical Care of Patients with Obesity^28^.

In patients with multimorbidity, early detection can guide treatment as well as slow progression, or potentially halt disease processes entirely. In this study, among young men there was a strong association between low testosterone and multimorbity. It is unlikely that end-stage disease was responsible for this finding, and thus it can be hypothesized that low testosterone may play an early, causal role in chronic disease processes. This warrants further research as it could change screening guidelines for testosterone deficiency, as well as have a significant impact on the rates of disease processes such as hypertension and diabetes in men.

Not surprisingly, nearly all research related to the influence of testosterone deficiency to potentiate risk for secondary muscle and metabolic dysfunction in men has been conducted within an aging-related context. However, the underlying changes in hormonal and metabolic dysregulation leading to multimorbidity should be regarded as a gradual continuous process throughout the lifespan, rather than a discrete outcome or event. Thus, further evaluation of the temporal sequence of these consequences is of particular importance not only for screening efforts to reduce chronic disease, but also for informing early, targeted interventions to treat declining TT or testosterone deficiency in men. In this regard, debate surrounding the cardiovascular safety of testosterone replacement therapy (TRT) persists because the Testosterone in Older Men with Mobility Limitations (TOM) trial was discontinued due to a higher prevalence of cardiovascular-related events in the TRT vs placebo groups^29^ (even though the study has received criticism primarily due to relatively poor classification of cardiovascular events^30^), and the findings of two large retrospective studies indicated increased cardiovascular disease risk in men receiving TRT^31,32^. While these findings raised initial concerns about cardiovascular safety of TRT, the aforementioned retrospective studies have received extensive scientific criticism^33^ and calls for retraction from numerous medical societies^34^. Moreover, they remain at odds with data from several recent meta-analyses that indicate TRT does not increase cardiovascular events in hypogonadal men^35–37^, and with findings of a very large retrospective study (n = 83,010 men with documented low T) that indicated TRT was associated with a 56% lower propensity matched all-cause mortality (Hazard Ratio [HR]: 0.44; 95%CI: 0.42–0.46) and 24–36% lower stroke risk (HR:0.64; 95% CI: 0.43 to 0.96) and MI risk (HR:0.76; 95% CI: 0.63 to 0.93), in comparison to untreated hypogonadal men^38^. It is also important to note that the recently published NIH-funded T-trials found no differences in cardiovascular-related or other adverse events among men receiving TRT vs placebo^39^, and there is also recent evidence that TRT is associated with lower mortality rates among diabetic men^40^.

Interestingly, despite that TT and muscle strength are known to be highly associated in men^41^, and that weakness is a known predictor of chronic disease and early mortality, our findings reveal an independent effect of muscle weakness and lower TT on risk for multimorbidity. Future research is needed to better understand the independent and joint effects of low TT and muscular weakness in young adulthood as a risk exposure for multimorbidity tracking into/throughout middle-age and older adulthood.

### Limitations

The design of this study was limited by the cross-sectional design of NHANES, which posed challenges in causal inference, especially with respect to reverse causation. Thus, we are unable to deduce whether low TT leads to higher odds of multimorbidity, or conversely, whether poor chronic health profiles lead to declines in TT levels. Moreover, we were unable to determine if other competing risks or unmeasured confounding (e.g., dietary habits, medication use) may have influenced the observed estimates. Future longitudinal studies are needed to better understand how declines in TT contribute to unhealthy aging, as well as the extent to which other chronic conditions (e.g., obesity, diabetes) may mediate the association between low TT, and multimorbidity and mortality. Third, we did not have access to sex hormone-binding globulin or free testosterone for these men, and thus we could not determine the differential contribution of these compared to TT for prediction of multimorbidity across the age spectrum. Lastly, we found no statistical differences in TT and testosterone deficiency between middle aged and older men, a finding that might be due to an underrepresentation of institutionalized older men.

## Conclusions

Our results suggest a much higher prevalence of testosterone deficiency occurs in men across the adult age-span than what has been previously reported, and that young and elderly men with testosterone deficiency exhibit a significantly higher multimorbidity risk than their eugonadal counterparts.
